# Intermittent Chaos in the CSTR Bray–Liebhafsky Oscillator-Specific Flow Rate Dependence

**DOI:** 10.3389/fchem.2020.560274

**Published:** 2020-10-23

**Authors:** Itana Nuša Bubanja, Ana Ivanović-Šašić, Željko Čupić, Slobodan Anić, Ljiljana Kolar-Anić

**Affiliations:** ^1^Faculty of Physical Chemistry, University of Belgrade, Belgrade, Serbia; ^2^Institute of Chemistry, Technology and Metallurgy, University of Belgrade, Belgrade, Serbia

**Keywords:** intermittent chaos, Bray–Liebhafsky oscillatory reaction, Lyapunov exponents, continuously-fed well-stirred tank reactor (CSTR), non-linear dynamics

## Abstract

Dynamic states with intermittent oscillations consist of a chaotic mixture of large amplitude relaxation oscillations grouped in bursts, and between them, small-amplitude sinusoidal oscillations, or even the quiescent parts, known as gaps. In this study, intermittent dynamic states were generated in Bray–Liebhafsky (BL) oscillatory reaction in an isothermal continuously-fed, well-stirred tank reactor (CSTR) controled by changes of specific flow rate. The intermittent states were found between two regular periodic states and obtained for specific flow rate values from 0.020 to 0.082 min^−1^. Phenomenological analysis based on the quantitative characteristics of intermittent oscillations, as well as, the largest Lyapunov exponents calculated from experimentally obtained time series, both indicated the same type of behavior. Namely, fully developed chaos arises when approaching to the vertical asymptote which is somewhere between two bifurcations. Hence, this study proposes described route to fully developed chaos in the Bray-Liebhafsky oscillatory reaction as an explanation for experimentally observed intermittent dynamics. This is in correlation with our previously obtained results where the most chaotic intermittent chaos was achieved between the periodic oscillatory dynamic state and stable steady state, generated in BL under CSTR conditions by varying temperature and inflow potassium iodate concentration. Moreover, it was shown that, besides the largest Lyapunov exponent, analysis of chaos in experimentally obtained intermittent states can be achieved by a simpler approach which involves using the quantitative characteristics of the BL reaction evolution, that is, the number and length of gaps and bursts obtained for the various values of specific flow rates.

## Introduction

The intermittent dynamic state is a chaotic state where two types of dynamic emerge in time, replacing each other stochastically. (Hilborn, 2004) The extent to which one type dominates the other can vary depending on the control parameter values. (Hilborn, 2004) In here considered case, intermittent dynamic state (also known as intermittent oscillations, intermittent chaos, or simply intermittency) (Pomeau and Manneville, 1980; Hilborn, 2004; Schuster and Just, 2005) represents a chaotic mixture of large amplitude relaxation oscillations grouped in bursts, and between them, there are small-amplitude sinusoidal oscillations or quiescent parts, known as gaps. This type of deterministic dynamic phenomenon may also be found in some complex chemical (Chopin-Dumas, 1978; Pomeau et al., 1981; Roux et al., 1981; Baier et al., 1989; Kreisberg et al., 1991; Strizhak and Menzinger, 1996; Vukojević et al., 2000; Kolar-Anić et al., 2004; Cadena et al., 2013; Čupić et al., 2014; Bubanja et al., 2016, 2017) and biochemical (Izhikevich, 2000a,b) reaction systems under conditions that do not have an equilibrium.

This study examined the appearance of intermittent chaos in the Bray–Liebhafsky (BL) reaction, which has been systematically investigated for decades by the Belgrade research group (Kolar-Anić et al., 2017). In this catalytic oscillatory reaction hydrogen peroxide is decomposed into water and oxygen in the presence of hydrogen and iodate ions (Bray, 1921; Bray and Liebhafsky, 1931).

$$\begin{array}{l} 2H_{2}O_{2}\;\to \;2H_{2}O\;+\;O_{2}\;\left(D\right) \end{array}$$

Hydrogen peroxide decomposition is the result of two complex pathways in which hydrogen peroxide acts either as a reducing (R) or as an oxidizing (O) agent:

$$\begin{array}{l} 2IO_{3}^{-}\;+\;2H^{+}+\;5H_{2}O_{2}\;\to \;I_{2}\;+\;6H_{2}O\;+\;5O_{2}\;\left(R\right) \end{array}$$

$$\begin{array}{l} I_{2}\;+\;5H_{2}O_{2}\;\to \;2IO_{3}^{-}\;+\;2H^{+}+\;4\;H_{2}O\;\left(O\right) \end{array}$$

Mentioned reactions (R) and (O) are complex and their mechanisms involve several intermediate species (Bray, 1921; Liebhafsky, 1931; Woodson and Liebhafsky, 1969; Furrow, 1985, 1987; Stanisavljev et al., 2013; Holló et al., 2014). In general, alternating increases and decreases of the intermediate species concentrations in time appear as a consequence of alternating dominations of reactions (R) and (O).

Taking into account that oscillatory dynamic states, the BL reaction can be realized either at relatively higher temperatures, where the potentiometric measurements are very complex or at lower temperatures where such experiments, lasting several days are difficult to control. Due to the fact that the intermittent chaos can be obtained only within a very narrow range of the control parameter values, it is not surprising that only a small number of scientific papers are dedicated to intermittent chaos in BL systems. To the best of our knowledge, only four papers considering intermittent phenomena in the BL system were found. Among them, two papers were published over 40 (Chopin-Dumas, 1978) and 20 years ago (Vukojević et al., 2000) while the remaining two (Bubanja et al., 2016, 2017) were published more recently.

In the most recent papers (Bubanja et al., 2016, 2017), intermittent dynamic states were experimentally generated in the BL reaction system realized in a continuously-fed well-stirred tank reactor (CSTR) under variations of temperature and inflow concentration of potassium iodate. These two last papers, clearly show that experimentally generated intermittent chaos can be analyzed, not only by calculations of the largest Lyapunov exponents (λ) from experimentally obtained oscillograms but also by the simple quantitative characteristics of the evolution of BL reaction, lengths of bursts, (packages of relaxing oscillations) and lengths of gaps between them (the periods of the sinusoid oscillations of relatively small amplitude when compared to relaxing oscillations). In this instance, a general problem that appears during the calculation of the largest Lyapunov exponents occurs from experimental noise in the signal, which cannot be avoided. Because of noise, even obviously regular oscillations give false-positive values of the largest Lyapunov exponents appearing as false chaotic states.

This study, building upon previous research (Bubanja et al., 2016, 2017), aimed to examine if there is a causality between the structure of intermittent chaos and the specific flow rate as the control parameter and whether this causality is consistent with these previous findings. For appropriate application in the characterization of relations among the different dynamic states, this study adapted previously established methods and techniques, which are described in the Results and Discussion of this paper.

## Experiment

**Figure 1:**
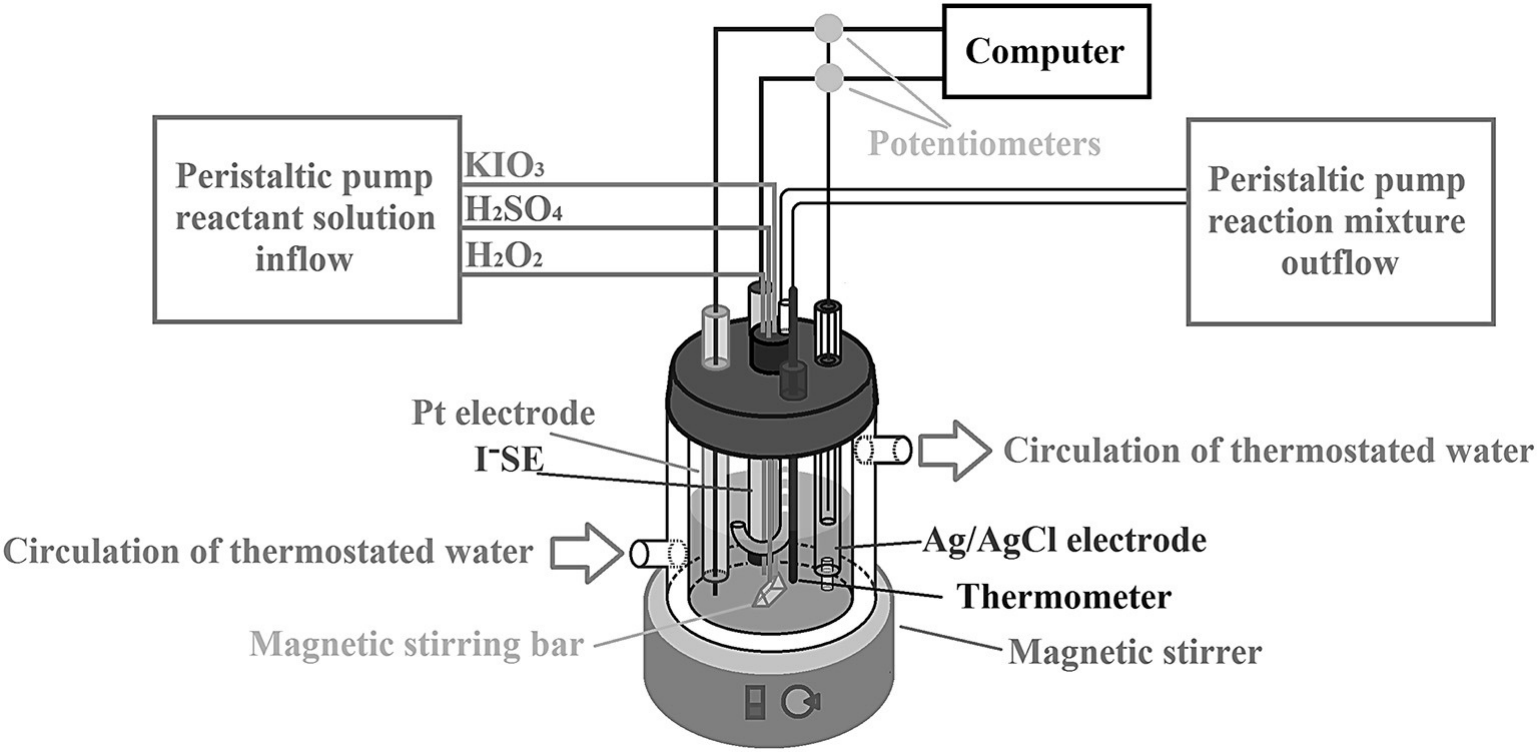
A schematic view of the experimental setup used for generating intermittent chaos in the BL system under CSTR conditions by varying the specific flow rate as a control parameter.

The BL reaction represents a decomposition of hydrogen peroxide into water and oxygen in the presence of iodate ions and acidic medium. In all our papers and here as well, as the source of iodate ions potassium iodate was used, and for setting up a desired acidity sulfuric acid was taken. Experiments were carried out at a constant temperature of (63.0 ± 0.1)^o^C, in a 50 mL (Metrohm reaction vessel, product number: EA876-20) glass CSTR vessel wrapped in a water recirculation jacket connected to a thermostat. For homogenization of the reaction mixture, a magnetic stirrer with a stirring speed of σ = 900 rpm and magnetic stirring bar (uniform triangular prism-shaped magnetic bar, length size 12 mm, and triangle side size 6 mm) covered with polytetrafluorethylen (PTFE) were used. The amounts of reactant species in the reactor were controlled by peristaltic pumps. Three channels were used to deliver solutions of the reactants KIO_3_, H_2_SO_4_, and H_2_O_2_ prepared in deionized water (18 MΩ·cm), and one channel of the other pump was used to remove the surplus volume of the reaction mixture through a U-shaped glass tube, to keep the constant volume of the reaction mixture (22.2 ± 0.2) mL. A potentiometric method was used to record the dynamic states of the BL reaction system, and a three-electrode system was applied. A double-junction Ag/AgCl was used as a reference electrode while working an iodide ion sensitive electrode (I^−^SE) and Pt electrode. Potential changes of Pt vs. Ag/AgCl and I^−^SE vs. Ag/AgCl during the time (well known in the literature as time series or oscillograms) were traced and recorded at the same time with an electrochemical instrument (PC-Multilab EH4 16-bit ADC) coupled with a personal computer. A schematic view of the experimental setup is given in Figure 1.

The experiment was performed first, by filling the reactor with reactants at the total flow rate of 15.0 mL/min, protecting the thermostat reaction vessel from light. Concentrations of stock solutions were chosen so that their inflow concentrations in the reactor were: [KIO_3_]_0_ = 0.035 M, [H_2_SO_4_]_0_ = 0.077 M, and [H_2_O_2_]_0_ = 0.240 M (M stands for mol·dm^−3^). After 1 min magnetic stirrer was turned on and set to stirring speed of 900 rpm. Then, after 2.95 min the total flow rate was set to 0.84 mL/min, and we switched on another pump for removing the surplus volume of the reaction mixture. The total flow rate of 0.84 mL/min corresponds to a specific flow rate of 0.038 min^−1^. This value was obtained by dividing the total flow rate (υ) with the total volume of the reaction mixture (V):

$$\begin{array}{l} j_{0}=\frac{\upsilon_{KIO_{3}}+\upsilon_{H_{2}SO_{4}}+\upsilon_{H_{2}O_{2}}}{V}=\frac{\upsilon }{V}=\frac{0.84\;mL/min}{22.2\;mL}\\ \;=0.038\;\min^{-1} \end{array}$$

Every following experiment was performed as an extension of the previous one with a decreased or increased total flow rate of reactants and, therefore, with decreased or increased specific flow rate. Between each experiment, the recording was paused for a period of three retention times (retention time represents the reciprocal value of the specific flow rate). This allowed the system to stabilize in a new dynamic state with a changed control parameter.

## Results and Discussion

**Figure 2:**
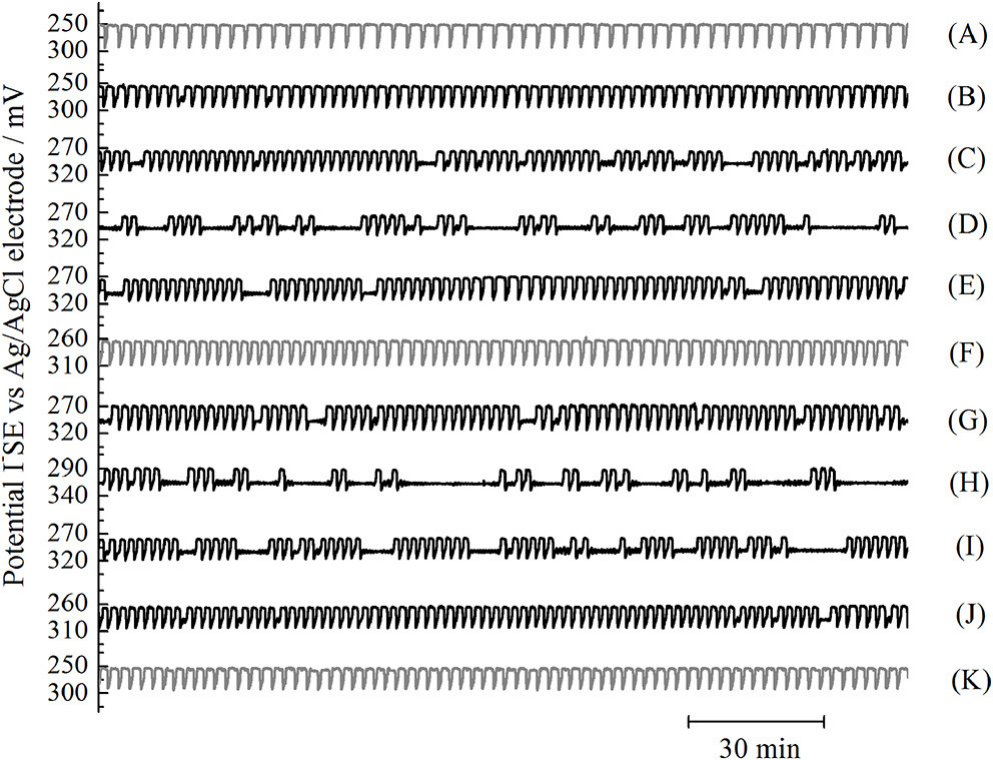
Transitions between periodic oscillatory dynamic states [**(A)** j_0_ = 0.095 min^−1^ **(F)**, j_0_ = 0.020 min^−1^, and **(K)** j_0_ = 0.101 min^−1^] via different intermittent states, obtained either by decreasing [**(B)** j_0_ = 0.077 min^−1^, λ = 34.8; **(C)** j_0_ = 0.064 min^−1^, λ = 81.6; **(D)** j_0_ = 0.061 min^−1^, λ = 95.1; **(E)** j_0_ = 0.038 min^−1^, λ = 68.4] or increasing [**(G)**, j_0_ = 0.038 min^−1^, λ = 63.4; **(H)** j_0_ = 0.064 min^−1^, λ = 92.7; **(I)** j_0_ = 0.072 min^−1^; **(J)** j_0_ = 0.077 min^−1^, λ = 67.6] specific flow rate. Data from 3 h of the time series (I^−^SE vs. Ag/AgCl potential over time) are shown (note: oscillograms were recorded over a longer period). Oscillograms were obtained for the following experimental conditions: [KIO_3_]_0_ = 0.035 M, [H_2_SO_4_]_0_ = 0.077 M, [H_2_O_2_]_0_ = 0.240 M, T = 63.0 ^o^C, σ = 900 rpm, while the specific flow rate was varied. For noise removal in Origin 8 application, Savitzky–Golay filtering was applied. Oscillograms in gray correspond to periodic oscillatory states, and those that are black represent intermittent dynamic states. Besides specific flow rates, values of largest Lyapunov exponent, λ are stated where applicable.

Under CSTR conditions in the considered range of specific flow rate, we found the chaotic mixture of the regular sustained large-amplitude relaxation oscillations grouped in bursts and small-amplitude irregular sinusoidal ones grouped in gaps. Chaotic intermittent nature of recorded time series is reflected in irregular and unpredictable lasting of both, bursts and gaps. Figure 2, shows the dynamic states of the BL system in CSTR under variation of specific flow rate (j_0_), which were monitored by potential changes of iodide ion sensitive (I^−^SE) vs. Ag/AgCl electrode over time. The first chaotic emergence of low-amplitude oscillations, which occurred when the specific flow rate decreased, shifting the system from a periodic oscillatory dynamic state (Figure 2A, gray oscillogram), denotes the emerging of an initial intermittent state (Figure 2B). This phenomenon appears when the defined value of the specific flow rate is slightly lower than the value where the periodic dynamic state was found. With further decreasing of specific flow rate the low-amplitude oscillations transform to the short gaps. Their number and duration increase at first and then decrease. Finally, the gaps disappear at a very low specific flow rate, when a periodic oscillatory dynamic state was again obtained (Figure 2F). As the specific flow rate increases, starting from the state given in Figure 2F, the whole scenario repeats, and the system reaches the periodic oscillatory dynamic state once more.

Thus, in here considered case where the specific flow rate is used as a control parameter, the intermittent chaos was observed between two periodic oscillatory states. When variations of temperature (Bubanja et al., 2016) and inflow concentration of potassium iodate (Bubanja et al., 2017) were used to generate intermittent oscillations in the BL system, intermittencies emerged between the periodic oscillatory state and stable steady state.

**Figure 3:**
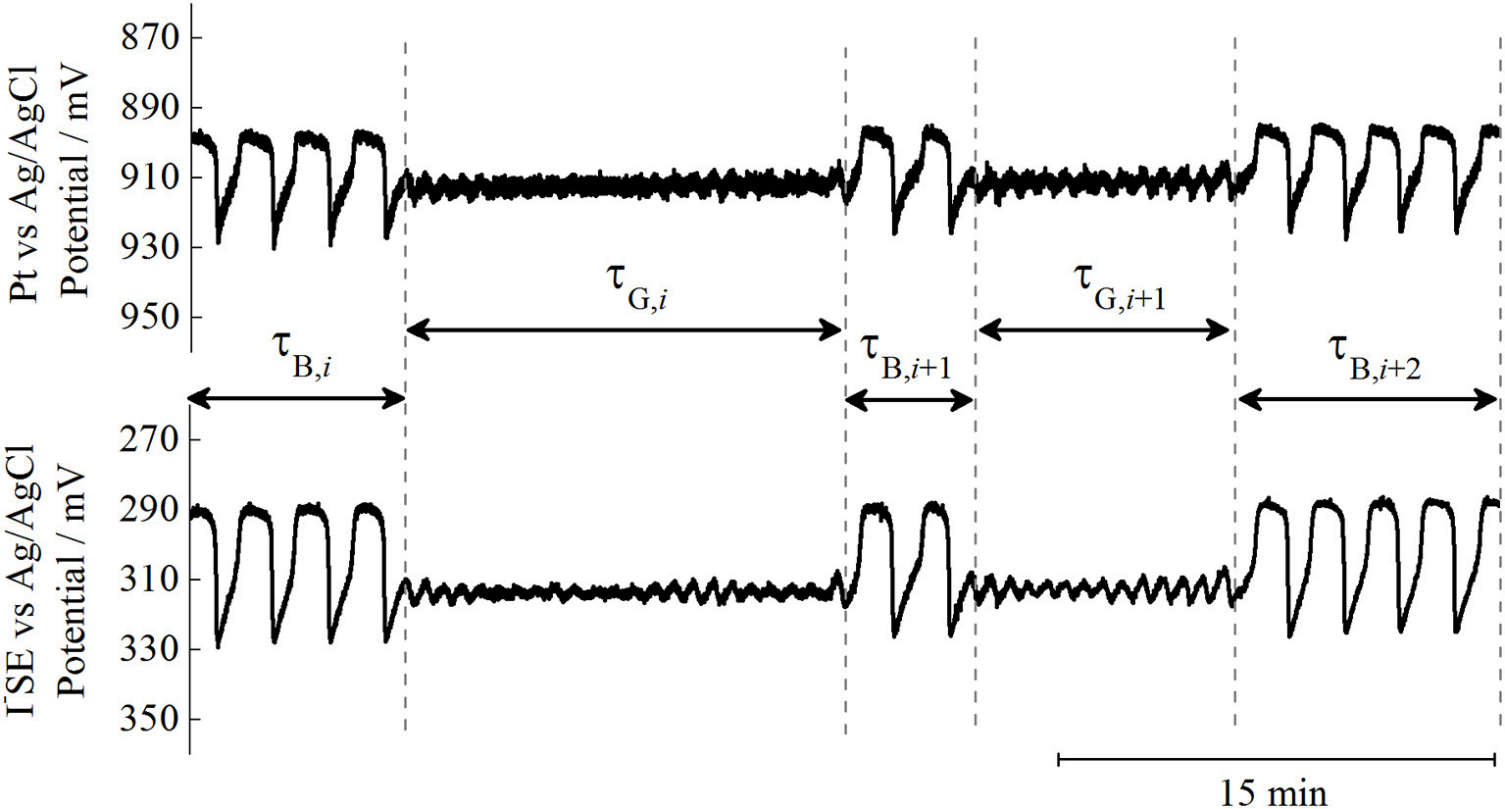
Potentiometric traces, recorded by Pt (upper) and I^−^SE (lower) electrodes vs. referent Ag/AgCl, over identical time intervals in order to illustrate different noise levels. Displayed oscillograms were obtained for the following experimental conditions: [KIO_3_]_0_ = 0.035 M, [H_2_SO_4_]_0_ = 0.077 M, [H_2_O_2_]_0_ = 0.240 M, T = 63.0^o^C, σ = 900 rpm and j_0_ = 0.068 min^−1^.

In our previous experiments only potential changes of Pt versus Ag/AgCl electrode were used to record dynamics of BL system (Bubanja et al., 2016, 2017). In the present study potential changes of I-SE versus Ag/AgCl electrode were monitored simultaneously with potential changes of Pt versus Ag/AgCl electrode. Our previous experiments identified potential changes of Pt vs. the Ag/AgCl electrode, which were used to record the dynamics of the BL system, in the present study the potential changes of I^−^SE vs. Ag/AgCl electrode were monitored simultaneously with potential changes of Pt vs. Ag/AgCl electrode. Figure 3 shows that the same segment of oscillograms obtained by Pt *vs*. Ag/AgCl and I^−^SE *vs*. Ag/AgCl electrodes for a specific flow rate of j_0_ = 0.068 min^−1^.

**Figure 4:**
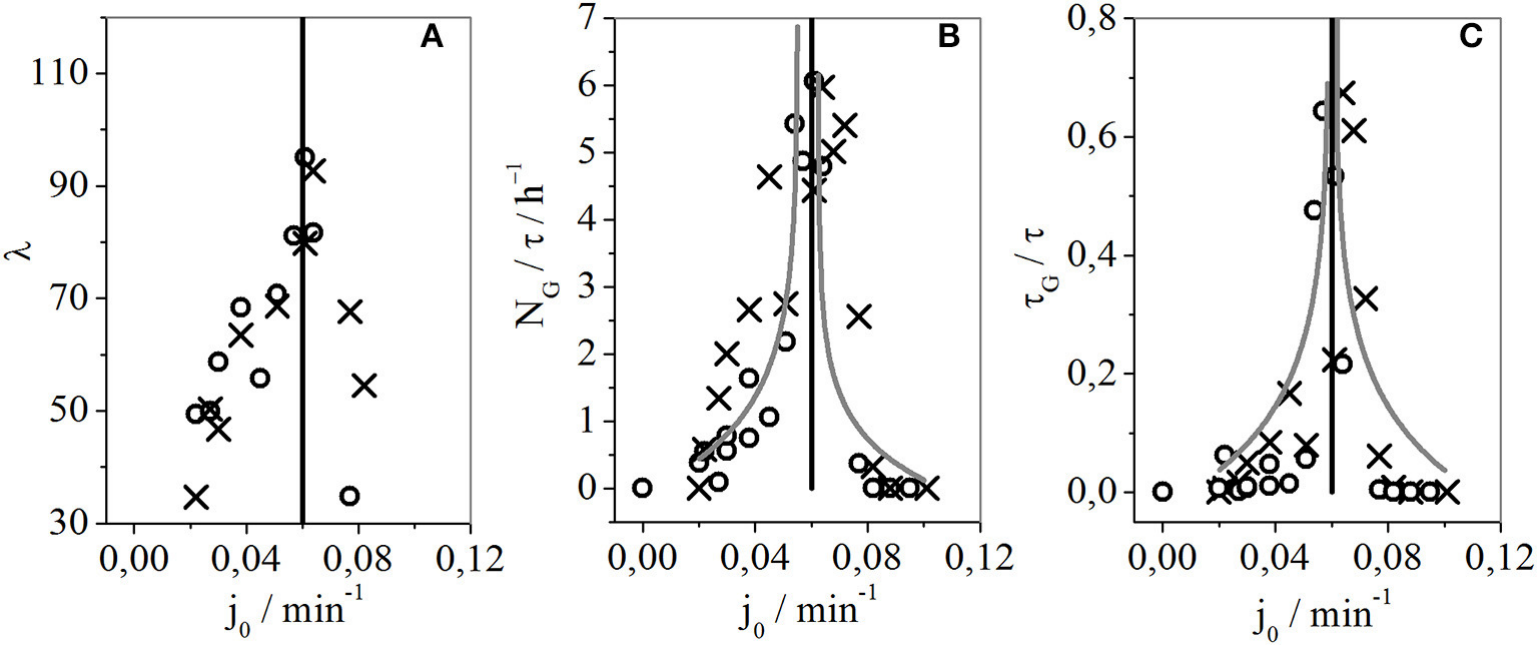
**(A)** Largest Lyapunov exponents, calculated from time series obtained by I^−^SE vs. Ag/AgCl electrode; **(B)** Number of gaps per unit of time (N_G_/τ, where τ is the duration of the experiment); **(C)** Fraction of time occupied by gaps (τ_G_/τ, where τ_G_ is the sum of gaps duration during one experimental recording). In all three cases, the control parameter is the specific flow rate. Cross-shaped symbols (×) and empty circles (°) correspond to the specific flow rate obtained during the increase and decrease directions, respectively.

In this system, where the iodide ion sensitive electrode (I^−^SE) and Pt electrode were used both together within the same reaction mixture, both potentiometric records of the bursts and gaps appear at the same time and have the same number of large amplitude oscillations per burst (Figure 3). However, it is noticeable that the potential signal traced with I^−^SE was less noisy than the one obtained when Pt was used as a working electrode. This is in accordance with our previous investigations of the regular periodic oscillations in BL reaction (Anić and Kolar-Anić, 1986; Anić et al., 1987). Since noise level is of crucial importance to any method of chaos quantification, the largest Lyapunov exponents were calculated from time-series obtained by the I^−^SE electrode. For the largest Lyapunov exponent calculations we applied the Wolf algorithm (Wolf et al., 1985). The Wolf algorithm was successfully used to quantify chaos when different chaotic dynamic states were obtained numerically (Ivanović-Šašić et al., 2011). Besides, even when applied to experimentally obtained intermittent chaos the Wolf algorithm showed a good correlation (Bubanja et al., 2016, 2017), considering the noise presence. Therefore, only the time series in which intermittencies were detected were used to calculate the largest Lyapunov exponents by the Wolf algorithm as a function of the control parameter (Figure 4A). Moreover, not all recorded intermittent states were appropriate for calculations of the largest Lyapunov exponent since in some long experiments technical problems emerged such as the drift in electrode potential after some time (probably due to unavoidable, slow reference electrode leakage).

As explained above, (Figure 2) by varying the control parameters, gaps gradually emerge from periodic oscillatory states due to the development of a new intermittent dynamic state. We noticed that variation of gap lengths (as well as burst lengths) over time is chaotic (irregular/unpredictable) and that somewhere in the middle of the region, for which the control parameter was varied, gaps become more dominant in the considered time frame than the bursts. In our previously published papers, the number of bursts per unit of time (N_B_/τ, where τ is the duration of the experiment) was used as a measure of chaos in intermittent dynamic states. Even though, the number of bursts and gaps per each time series are almost identical, in presented case it is more correct to use number of gaps per unit of time since chaotic states are generated by inserting the increasing number of gaps between burst packages made of regular periodic oscillations. Taking this into consideration, besides the largest Lyapunov exponents (λ), the measure of chaos in considered intermittent state is obviously the number of gaps per unit of time (N_G_/τ). Therefore, the calculated largest Lyapunov exponents, as a function of specific flow rate (Figure 4A), were compared with the number of gaps per unit of time as a function of the same control parameter (Figure 4B). The functional dependence of the largest Lyapunov exponents *vs*. the specific flow rate follows the functional dependence of the number of gaps per time *vs*. the specific flow rate. Moreover, a significant level of linear correlation among the largest Lyapunov exponent and the number of gaps per unit of time was confirmed (see Supplementary Material).

As seen in Figure 4B, by further analyzing the results of the experiment we can see that somewhere in the middle of the region for which the control parameter was varied (roughly around 0.06 min^−1^), the dynamic states of the considered reaction system tend to achieve “fully developed chaos.” However, as we are closer to the mentioned fully developed chaotic state, the system is more sensitive to control parameters and the realization of these states is more difficult. Consequently, in the vicinity of the vertical asymptote, which is presented in Figures 4A–C, the dispersion of experimental points is the most evident. In this region, some experimental results could not be analyzed by the Wolf method using the largest Lyapunov exponents. To capture and record the selected dynamic state the experiment should be very long, which is serious experimental problem. It is difficult to keep the reaction system in so sensitive dynamic state for several days. Nevertheless, the shape of the functional dependence between the largest Lyapunov exponents and specific flow rate matches the general observation of the obtained chaotic dynamics within a narrow range of the flow rates with the intermittent dynamic states.

**Figure 5:**
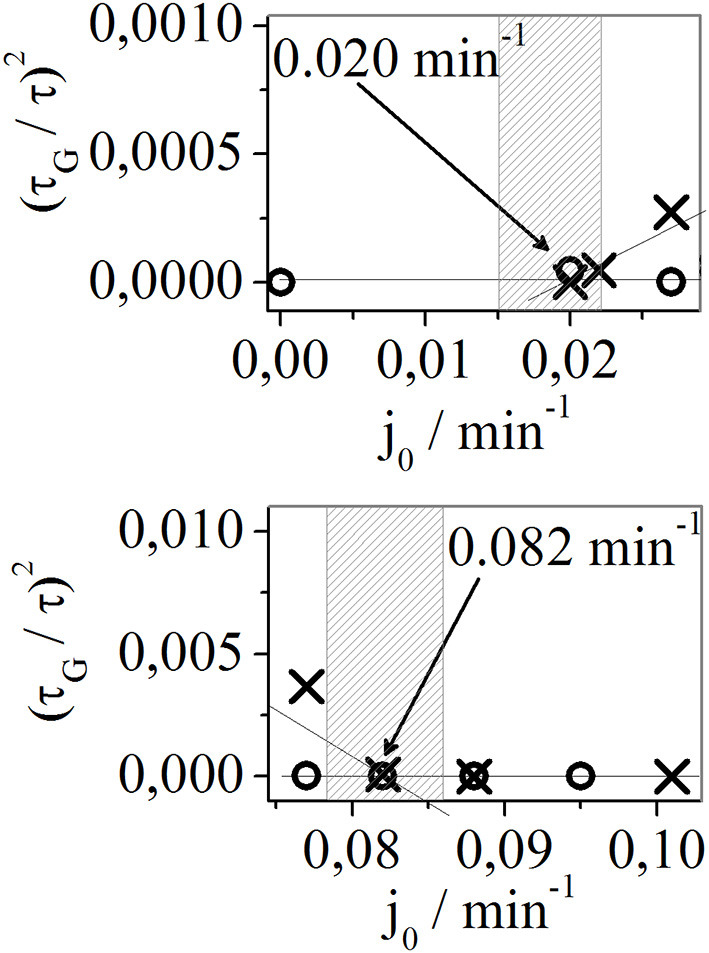
The (τ_G_/τ)^2^ as a function of the specific flow rate. The bifurcation points are roughly estimated from the linear relationship between (τ_G_/τ)^2^ and the specific flow rate in the vicinity of the bifurcation point, marked with arrows. The estimated/expected bifurcation points are shown in the shaded area. As in Figure 4, cross-shaped symbols (×) and empty circles (°) correspond to the specific flow rate obtained during the increase and decrease directions, respectively. Only results around critical values are shown.

Moreover, we wanted to define the boundaries of the region of specific flow rates with intermittent dynamic states. However, the task of determining the bifurcation points between the periodic and intermittent oscillations is difficult. In the vicinity of bifurcation, only one short gap might appear just once after several days. Therefore, we decided to look for bifurcation points in analogy with other reaction systems where transitions between qualitative different dynamic states under the changes of control parameter have not caused sudden or unexpected transitions between them, similarly to the appearance of the limit cycle from the stable steady state through supercritical Andronov-Hopf bifurcation. In this study, the packages of small amplitude oscillations grouped in gaps emerge gradually from the regular oscillations described by the large amplitudes. Therefore, the fraction of time occupied by gaps (τ_G_/τ) slowly increases from zero in the vicinity of the bifurcation point. This reflects the slow emerging limit cycle in Andronov-Hopf bifurcation as a function of the control parameter. Hence, we presented (τ_G_/τ)^2^ as a function of the control parameter of the specific flow rate (Figure 5) and analyzed the linear relationship around critical values.

From Figure 5 it is very difficult to evaluate the exact critical values of specific flow rates that correspond to bifurcation points. We can only estimate that those bifurcation points occur around 0.020 and 0.082 min^−1^. This roughly estimated region, in which the appearance of the bifurcation point could be expected, is indicated by the shaded area in Figure 5. The bifurcation point close to 0.020 min^−1^ corresponds to the bifurcation representing the occurrence of gaps from regular periodic oscillations, obtained for lower specific flow rate values. Bifurcation close to 0.082 min^−1^ corresponds to the occurrence of gaps from regular periodic oscillations, obtained for higher specific flow rate values. Moreover, no hysteresis was obtained when experiments were performed with decreasing/increasing specific flow rates.

**Figure 6:**
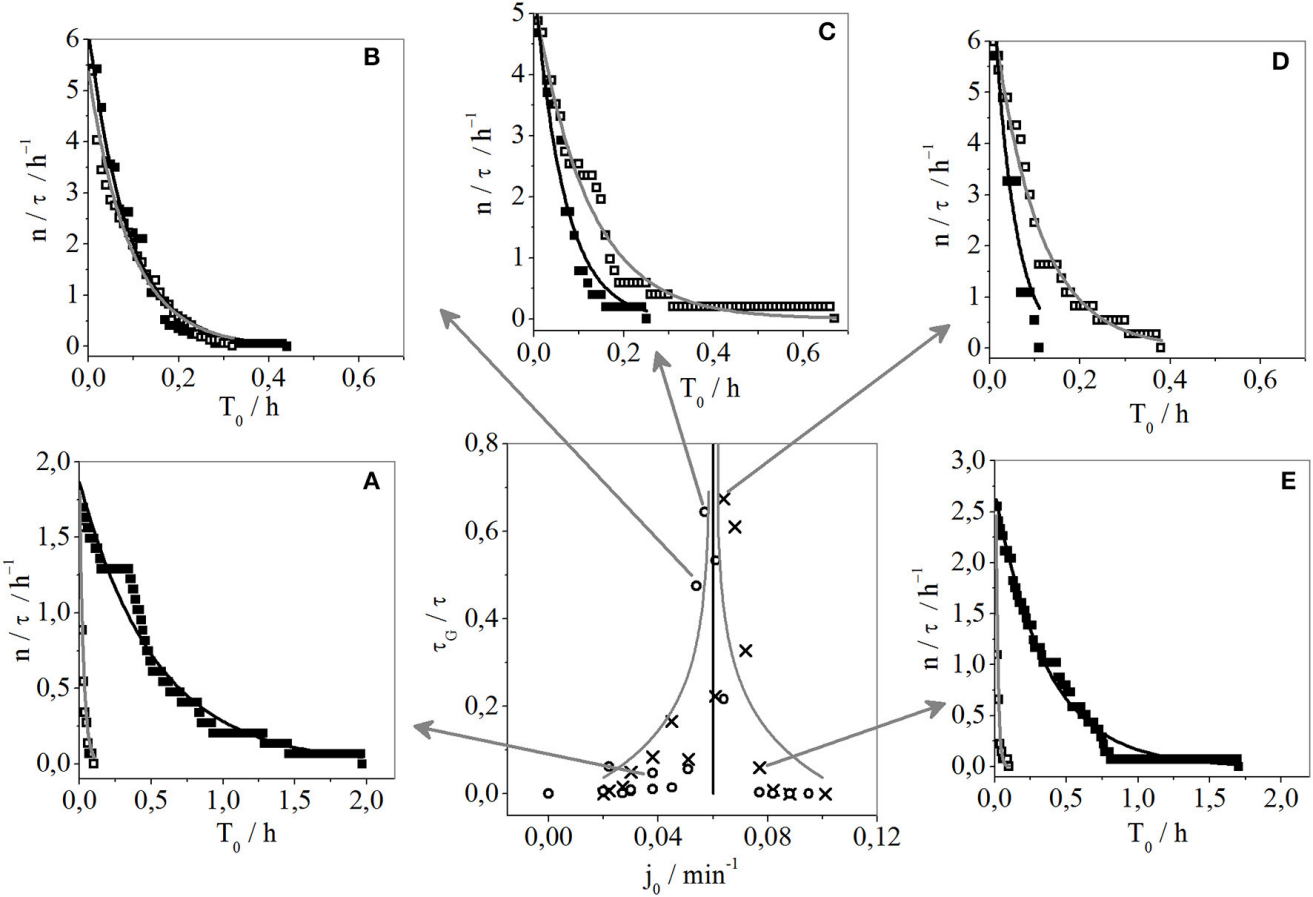
Relative number of burst or gap packages per time τ (n/τ) with duration *l* > T_0_ (N(*l* > T_0_)/τ) or, simply (n/τ) where *n* = N(*l* > T_0_) obtained for selected specific flow rate experiments (see figure in the middle where fraction of time occupied by gaps (τ_G_/τ) as a function of specific flow rate is presented) denoted by arrows: **(A)** 0.038 min^−1^, **(B)** 0.054 min^−1^, **(C)** 0.057 min^−1^, **(D)** 0.064 min^−1^, and **(E)** 0.077 min^−1^. The number of burst packages longer than T_0_ are marked with black squares and fitted with a black curve, while the number of gaps for *l* > T_0_ are marked with empty squares and fitted with gray curves.

As observed from Figure 2, in all the intermittent states the lengths of individual gaps and bursts are both chaotically distributed. However, in accordance with our previous studies in the quantitative analysis of intermittent chaos, we can see that by indicating the number of burst and gap packages whose duration is longer than some time, denoted as T_0_, the regularity in the distribution of the duration of burst and gap packages, per one experiment, can be observed (Figure 6). It should be stressed that relative values of the number of bursts/gaps longer than some T_0_ in the unit of time (N(*l* > T_0_)/τ) or, simply (n/τ) where *n* = N(*l* > T_0_) was used, and since the graphs for time series of very different overall lengths ought to be compared. Relative values (n/τ) were obtained by dividing the number of bursts/gaps by the total time of the experimental recording (τ).

The central graph of Figure 6 shows the fraction of time occupied by gaps as a function of the specific flow rate. The arrows indicate the results that were used to calculate the relative number of bursts and gaps longer than T_0_ (Figures 6A–E). The central graph in Figure 6 uses the same symbols as Figure 4C to describe the results during an increase or decrease of the specific flow rate. Replicating the trends obtained in a previous study by Dubois et al. (1983), where exponential dependence between some time T_0_ and the number of laminar phase intervals (gaps or bursts) longer than T_0_ was used as an indicator of type III intermittency, we applied less complex exponential dependence, to our results. To fit the results shown in Figures 6A–E, mathematical form y = *p*·exp(*q*·x) was used, where y and x stand for n/τ, and T_0_ respectively, while *p* and *q* are parameters used for optimization and their optimal values are given in Table 1, which includes the values of the adjusted R-squares. The letters B and G are given in the subscript of the p, q parameters, and adjusted R-squares (*R*^2^) to indicate from the data from which each fitting was performed. Namely, values with B in subscript were obtained using the number of burst packages whose duration is longer than T_0_. The values with G in subscript correspond to the number of gap packages with a duration longer than T_0_. As can be seen from Table 1, the mentioned mathematical form was convenient for all shown cases in Figure 6, as well as for both bursts and gaps per case, but with different values for the fitted parameters. Based on the high values of the adjusted coefficient of determination or adjusted R-square (the lowest was 0.946 and the highest was 0.994) we can conclude that the exponential equation fitted data well. That said, in case (Figure 6D) the adjusted *R*-square for q_B_ was slightly lower (at 0.897), as a consequence of the very short duration of this experimental recording and the relatively small number of bursts.

**Table 1:** Values of fit parameters (q, p) and adjusted *R*-square (*R*^2^), obtained for results given in Figure 6 from (A–E).

**Case**	**j_**0**_/min^**−1**^**	**q_**B**_**	**q_**G**_**	**q_**B**_/q_**G**_**	**p_**B**_**	**p_**G**_**	**${\text{R}}_{\text{B}}^{2}$**	**${\text{R}}_{\text{G}}^{2}$**
(A)	0.038	−1.889	−36.79	0.0514	1.186	1.806	0.971	0.954
(B)	0.054	−11.348	−10.868	1.044	6.153	5.354	0.978	0.983
(C)	0.057	−14.961	−8.419	1.777	5.561	5.279	0.946	0.975
(D)	0.064	−21.182	−9.975	2.124	8.018	6.908	0.897	0.979
(E)	0.077	−2.682	−73.823	0.0363	2.693	5.147	0.986	0.994

*Letter B in subscript corresponds to results obtained by fitting the number of burst packages whose duration was longer than T_0_, while letter G is related to the number of gap packages with duration longer T_0_*.

The ratio between fit parameters q for bursts (q_B_) and gaps (q_G_) in the same experiment, should reveal whether the black curve is “above” or “below” the gray (Table 1). However, regardless of the relative position of the black and gray curves, this was an instance of intermittent chaos. Moreover, even when q_B_ and q_G_ are nearly equal intermittent chaos also occurs. However, a higher ratio of q_B_/q_G_ indicates a higher level of complexity. This quantity could be a candidate for identifying the position of vertical asymptote with respect to the control parameter (Figure 4), which was expected to have low absolute values for q_G_ and high for q_B_, meaning that the ratio would tend to infinity, which would correspond to fully developed chaos. The relative positions of exponential curves can be used to describe the dominant state in the experimentally observed intermittent states. Namely, in intermittent states where lengths of gaps are short, and bursts dominate (Figures 6A,E), the exponential curve of bursts is above the corresponding curve for gaps, so the q_B_/q_G_ ratio is <1 [q_B_/q_G_ = 0.0514 (A), and q_B_/q_G_ = 0.0363 (E)], meaning it is opposite when the gaps start to dominate over bursts [Figure 6 q_B_/q_G_ = 1.777 (C), q_B_/q_G_ = 2.124 (D)]. From the results seen in Figure 6B, it could be concluded that both bursts and gaps are equally dominant in the intermittent state since q_B_/q_G_ is almost 1 (q_B_/q_G_ = 1.044). Thus, the method proposed by Dubois et al. (1983) is one way of quantifying the regularity seen in Figure 4C. That is, in both bifurcation points, when gaps emerge their fraction gradually increases while approaching a vertical asymptote, where they start to dominate. There, the dynamic states approach fully developed chaos, which is quantified by the largest Lyapunov exponents Figure 4A. The number of gaps (Figure 4B), the fraction of time occupied by gaps (Figure 4C), follow these overall dynamics. These results, which provide insights into the evolution of dynamic states, are in accordance with the known fact that the route to chaos can be realized through intermittent oscillations. Even though any intermittent state is a chaotic one, this study has shown that this tendency and increasing complexity occurs somewhere in the middle, among these two periodic oscillatory states (Nicolis and Prigogine, 1989; Ivanović-Šašić et al., 2011).

## Conclusions

This study examined a mixture of two types of dynamic state (bursts and gaps), which were chaotically combined to create a new intermittent dynamic state. This new intermittent state was experimentally generated in the Bray-Liebhafsky (BL) oscillatory chemical reaction in CSTR under variations of the specific flow rate, using the following experimental conditions: [KIO_3_]_0_ = 0.035 M, [H_2_SO_4_]_0_ = 0.077 M, [H_2_O_2_]_0_ = 0.240 M, σ = 900 rpm, T = 63.0^o^C. We observed intermittent dynamic states between these two regular periodic oscillatory states. By analyzing the bifurcation points, we found that they emerge in the following range of specific flow rates, between around 0.020 and 0.082 min^−1^.

In the intermittent region, chaotic complexity arises from both bifurcations, approaching a vertical asymptote that is somewhere between these flow rates, indicating a potential route to generating fully developed chaos. Hence, this study proposes that described route to fully developed chaos in the Bray-Liebhafsky oscillatory reaction is an explanation for experimentally observed intermittent dynamics.

This experiment obtained similar results to other previous studies, in which temperature and the inflow concentration of potassium iodate were taken as the control parameters (Bubanja et al., 2016, 2017). If there is a basin where the experimentally intermittent chaos is obtained, changes of any control parameter will govern the system to the closest periodic state or stable steady state. Consequently, future studies could adopt similar methods for the examination and quantification of the intermittent dynamic states.

This study showed that experimentally generated intermittent chaos can be analyzed, not only by calculations of the largest Lyapunov exponents (λ) from experimentally obtained oscillograms but also by simple quantitative analysis of the characteristics of the evolution of BL reaction, including the length of bursts (packages of relaxing oscillations), and the lengths of gaps (between the periods of the sinusoid oscillations of relatively small amplitude, when compared to relaxing oscillations).

## Data Availability Statement

The raw data supporting the conclusions of this article will be made available by the authors, without undue reservation.

## Author Contributions

INB performed the Bray-Liebhafsky experiments in CSTR. AI-Š calculated the Lyapunov exponents from the oscillograms obtained by the experiment. All authors created and developed this experiment, including the phenomenological analysis of the recorded intermittencies, and contributed to its conclusions.

## Conflict of Interest

The authors declare that the research was conducted in the absence of any commercial or financial relationships that could be construed as a potential conflict of interest.

## References

[B1] Anić S. Kolar-Anić Lj. (1986). The oscillatory decomposition of H_2_O_2_ monitored by the potentiometric method with Pt and Ag^+^/S^2−^ indicator electrode. Ber. Bunsenges. Phys. Chem. 90, 1084–1086. 10.1002/bbpc.19860901131

[B2] Anić S. Mitić D. Ćurčija M. (1987). The Bray-Liebhafsky reaction. III. Oscillatory decomposition of H_2_O_2_ in the presence of comparatively high acidity. J. Serb. Chem. Soc. 52, 575–579.

[B3] Baier G. Wegmann K. Hudson J. L. (1989). An intermittent type of chaos in the Belousov-Zhabotinsky reaction. Phys. Lett. A 141, 340–345. 10.1016/0375-9601(89)90061-3

[B4] Bray W. C. (1921). A periodic reaction in homogeneous solution and its relation to catalysis. J. Am. Chem. Soc. 43, 1262–1267. 10.1021/ja01439a007

[B5] Bray W. C. Liebhafsky H. A. (1931). Reactions involving hydrogen peroxide, iodine and iodate ion. I. Introduction. J. Am. Chem. Soc. 53, 38–44. 10.1021/ja01352a006

[B6] Bubanja I. N. Ivanović-Šašić A. Čupić Ž. Anić S. Kolar-Anić Lj. (2017). Intermittent chaos in the Bray-Liebhafsky oscillator. Dependence of dynamic states on the iodate concentration. Russ. J. Phys. Chem. A 91, 2525–2529. 10.1134/S0036024417130076

[B7] Bubanja I. N. Maćešić S. Ivanović-Šašić A. Čupić Ž. Anić S. Kolar-Anić Lj. (2016). Intermittent chaos in the Bray-Liebhafsky oscillator. Temperature dependence. Phys. Chem. Chem. Phys. 18, 9770–9778. 10.1039/C6CP00759G 27001164

[B8] Cadena A. Barragan D. Agreda J. (2013). Bursting in the Belousov-Zhabotinsky reaction added with phenol in a batch reactor. J. Braz. Chem. Soc. 24, 2028–2032. 10.5935/0103-5053.20130254

[B9] Chopin-Dumas J. (1978). Diagramme d'etat de la reaction de Bray. C. R. Acad. Sci. 287C, 553–555.

[B10] Čupić Ž. Kolar-Anić Lj. Anić S. Maćešić S. Maksimović J. Pavlović M. (2014). Regularity of intermittent bursts in Briggs-Rauscher oscillating systems with phenol. Helv. Chim. Acta 97, 321–333. 10.1002/hlca.201300178

[B11] Dubois M. Rubio M. A. Berge P. (1983). Experimental evidence of intermittencies associated with a subharmonic bifurcation. Phys. Rev. Lett. 51, 1446–1449. 10.1103/PhysRevLett.51.1446

[B12] Furrow S. (1987). Reactions of iodine intermediates in iodate-hydrogen peroxide oscillators. J. Phys. Chem. 91, 2129–2135. 10.1021/j100292a031

[B13] Furrow S. D. (1985). Chemical oscillators based on iodate ion and hydrogen peroxide, in Oscillations and Traveling Waves in Chemical Systems, eds. Field R. J. Burger M. (New York, NY: Wiley), 171–192.

[B14] Hilborn R. C. (2004). Chaos and Nonlinear Dynamics: An Introduction for Scientists and Engineers, 2nd Edn. Oxford: Oxford University Press.

[B15] Holló G. Kály-Kullai K. Lawson T. B. Noszticzius Z. Wittmann M. Muntean N.. (2014). HOI versus HOIO selectivity of a molten-type AgI electrode. J. Phys. Chem. A 118, 4670–4679. 10.1021/jp504052w 24892210

[B16] Ivanović-Šašić A. Marković V. M. Anić S. R. Kolar-Anić Lj. Z. Čupić Ž. D. (2011). Structures of chaos in open reaction systems, Phys. Chem. Chem. Phys. 13, 20162–20171. 10.1039/c1cp22496d 21993658

[B17] Izhikevich E. M. (2000a). Neural excitability, spiking and bursting. Int. J. Bifurcat. Chaos 10, 1171–1266. 10.1142/S0218127400000840

[B18] Izhikevich E. M. (2000b). Subcritical elliptic bursting of Bautm type. SIAM J. Appl. Math. 60, 503–535. 10.1137/S003613999833263X

[B19] Kolar-Anić Lj. Anić S. Čupić Ž. Ivanović-Šašić A. Pejić N. Blagojević S. I. (2017). Oscillating reactions, in Encyclopedia of Physical Organic Chemistry, Volume 2, Part 2 Organic Reactions and Mechanisms, ed. Wang Z. Wille Z, U. Juaristi U, E. (New York, NY: Wiley), 1127–1222.

[B20] Kolar-Anić Lj. Vukojević V. Pejić N. Grozdić T. Anić S. (2004). Deterministic chaos in open well-stirred Bray-Liebhafsky reaction system in: Expermimental Chaos, ed Boccaletti S. (Melville, NY: American Institute of Physics, AIP conference proceedings), 742, 3–8.

[B21] Kreisberg K. McCormick W. D. Swinney H. L. (1991). Experimental demonstration of subtleties in subharmonic intermittency. Phys. D 50, 463–477. 10.1016/0167-2789(91)90010-7

[B22] Liebhafsky H. A. (1931). Reactions involving hydrogen peroxide, iodine and iodate ion. IV. The oxidation of iodine to iodate ion by hydrogen peroxide. J. Am. Chem. Soc. 53, 2074–2090. 10.1021/ja01357a006

[B23] Nicolis G. Prigogine I. (1989). Exploring Complexity. New York, NY: Freeman.

[B24] Pomeau Y. Manneville P. (1980). Intermittent transition to turbulence in dissipative dynamical systems. Commun. Math. Phys. 74, 189–197. 10.1007/BF01197757

[B25] Pomeau Y. Roux J. C. Rossi A. Bachelart S. Vidal C. (1981). Intermittent behavior in the Belousov-Zhabotinsky reaction. J. Phys. Lett. 42, 271–273. 10.1051/jphyslet:019810042013027100

[B26] Roux J. C. Rossi A. Bachelart S. Vidal C. (1981). Experimental observations of complex dynamical behavior during a chemical reaction. Phys. D 2, 395–403. 10.1016/0167-2789(81)90018-X

[B27] Schuster H. G. Just W. (2005). Deterministic Chaos - An Introduction, 4th Edn. Weinheim: Wiley-VCH.

[B28] Stanisavljev D. R. Milenković M. C. Popović -Bijelić A. D. Mojović M. D. (2013). Radicals in the Bray-Liebhafsky oscillatory reaction. J. Phys. Chem. A 117, 3292–3295. 10.1021/jp402381b 23577613

[B29] Strizhak P. Menzinger M. (1996). Nonlinear dynamics of the BZ reaction: a simple experiment that illustrates limit cycles, chaos, bifurcations, and noise. J. Chem. Educ. 73, 868–873. 10.1021/ed073p868

[B30] Vukojević V. Anić S. Kolar-Anić Lj. (2000). Investigation of dynamic behavior of the Bray–Liebhafsky reaction in the cstr. Determination of Bifurcation Points. J. Phys. Chem. A 104, 10731–10739. 10.1021/jp001165x

[B31] Wolf A. Swift J. B. Swinney H. L. Vastno J. A. (1985). Determining Lyapunov exponents from a time series. Phys. D. 16, 285–317. 10.1016/0167-2789(85)90011-9

[B32] Woodson H. J Liebhafsky H. A (1969). Pulses in iodide concentrations during the peroxide decomposition of hydrogen peroxide. Nature 224:690 10.1038/224690a0

