# Supplementary material for: Intermittent Chaos in the CSTR Bray–Liebhafsky Oscillator-Specific Flow Rate Dependence
Source: Front Chem. 2020 Oct 23;8:560274. doi: 10.3389/fchem.2020.560274 (PMC7645043; doi:10.3389/fchem.2020.560274)
Supplement: Supplementary file 1 [file Data_Sheet_1.PDF]

Intermittent Chaos in the CSTR Bray–Liebhafsky oscillator

# - Specific flow rate dependence – Supplementary material

I. N. Bubanja, A. Ivanović-Šašić, Ž. Čupić, S. Anić, and Lj. Kolar-Anić

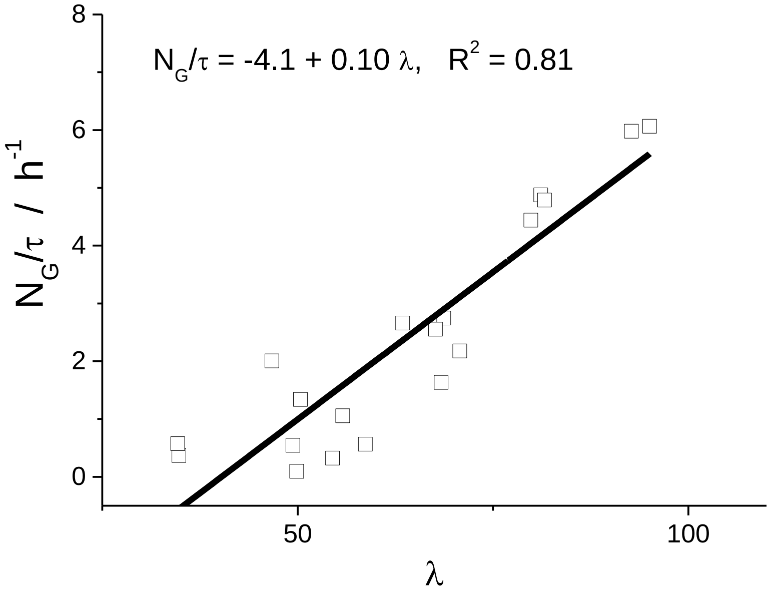

Figure S.1 Relative number of gaps over time as a function of the largest Lyapunov exponent λ. Complete set of data is used for linear data fit.

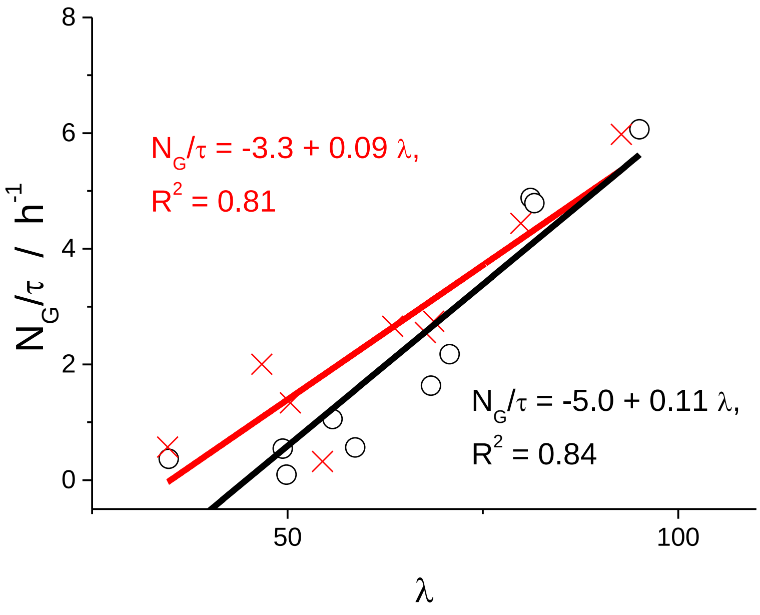

Figure S.2 Relative number of gaps over time as a function of the largest Lyapunov exponent λ. Separate data sets were used for linear data fits, one for experiments during increase (×), and the other during decrease (○) of the specific flow rate j_0 as the control parameter.
